# *APEX1* Polymorphisms and Neuroblastoma Risk in Chinese Children: A Three-Center Case-Control Study

**DOI:** 10.1155/2019/5736175

**Published:** 2019-06-25

**Authors:** Jiabin Liu, Wei Jia, Rui-Xi Hua, Jinhong Zhu, Jiao Zhang, Tianyou Yang, Peng Li, Huimin Xia, Jing He, Jiwen Cheng

**Affiliations:** ^1^Department of Pediatric Surgery, Guangzhou Institute of Pediatrics, Guangzhou Women and Children's Medical Center, Guangzhou Medical University, Guangzhou, 510623 Guangdong, China; ^2^Department of Oncology, The First Affiliated Hospital of Sun Yat-sen University, Guangzhou, 510080 Guangdong, China; ^3^Department of Clinical Laboratory, Molecular Epidemiology Laboratory, Harbin Medical University Cancer Hospital, Harbin, 150040 Heilongjiang, China; ^4^Department of Pediatric Surgery, The First Affiliated Hospital of Zhengzhou University, Zhengzhou, 450052 Henan, China; ^5^Department of Pediatric Surgery, The Second Affiliated Hospital of Xi'an Jiaotong University, Xi'an, 710004 Shaanxi, China

## Abstract

Neuroblastoma is a life-threatening extracranial solid tumor, preferentially occurring in children. However, its etiology remains unclear. *APEX1* is a critical gene in the base excision repair (BER) system responsible for maintaining genome stability. Given the potential effects of *APEX1* polymorphisms on the ability of the DNA damage repair, many studies have investigated the association between these variants and susceptibility to several types of cancer but not neuroblastoma. Here, we conducted a three-center case-control study to evaluate the association between *APEX1* polymorphisms (rs1130409 T>G, rs1760944 T>G, and rs3136817 T>C) and neuroblastoma risk in Chinese children, consisting of 469 cases and 998 controls. Odds ratio (OR) and 95% confidence intervals (CIs) were calculated to evaluate the associations. No significant association with neuroblastoma risk was found for the studied *APEX1* polymorphisms in the single locus or combination analysis. Interestingly, stratified analysis showed that rs1130409 GG genotype significantly reduced the risk of tumor in males. Furthermore, we found that carriers with 1-3 protective genotypes had a lower neuroblastoma risk in the children older than18 months and male, when compared to those without protective genotypes. In summary, our data indicate that *APEX1* gene polymorphisms may have a weak effect on neuroblastoma susceptibility. These findings should be further validated by well-designed studies with larger sample size.

## 1. Introduction

Neuroblastoma is the most frequently diagnosed extracranial pediatric malignancy in children younger than 12 months, which accounts for almost 10% of all pediatric malignancies and 15% of pediatric cancer-related deaths [1]. Neuroblastoma is a highly heterogeneous tumor with a wide range of clinical symptoms. For instance, some patients with innocent tumors have spontaneous regression, while others have poor prognosis even after receiving intensive treatment because of the distant metastasis [2]. Overall, neuroblastoma can be classified into low-, intermediate-, and high-risk groups depending on the clinical feature, pathological phenotypes, and prognostic factors [3]. Despite the great progresses made in multimode treatments for neuroblastoma, the survival rate remains unsatisfying. The overall 5-year survival rate is around 70%; however, the survival rate for high-risk patients is lower than 40% [4]. This poor prognosis may be partially attributed to widespread metastasis at the time of diagnosis [5].

Familial neuroblastoma with driver germline mutation is rare, accounting for approximately 1-2% of all cases [6]. The genetic alterations in the *PHOX2B* and *ALK* genes play an important role in familial neuroblastoma [7, 8]. However, the genetic mechanism of the sporadic neuroblastoma is poorly understood. Some environmental factors have been proposed as potential risk factors, such as maternal medication use, dwelling condition, childhood infections, conception, and pregnancy exposures [9, 10]. To date, a direct link between neuroblastoma and environmental factors is still missing. Excitingly, the progresses in the understanding of human genome and genotyping technologies have made the genome-wide association study (GWAS) a powerful tool to study inherited genetic variations, which are associated with complex human diseases (e.g., cancer) [11]. The first neuroblastoma GWAS was performed by Maris et al. in 2008. They found that *CASC15* gene polymorphisms were significantly related to neuroblastoma susceptibility [12]. Later on, the same group reported that common variants in *BARD1* gene were associated with high-risk neuroblastoma [13], and polymorphisms within *DUSP12*, *DDX4*, *IL31RA*, and *HSD17B12* were associated with low-risk neuroblastoma [14]. In 2011, Wang et al. demonstrated that *LMO1* gene polymorphisms could alter the neuroblastoma susceptibility [15]. Diskin et al. proved that polymorphisms in *LIN28B* and *HACE1* genes were also able to alter susceptibility to neuroblastoma [16]. Furthermore, the association of the polymorphisms in *TP53* [17] and *MMP20* [18] genes with the neuroblastoma risk was also discovered by recent GWASs successively. In addition, candidate gene approaches have been useful in identifying potential variants associated with neuroblastoma. For instance, Capasso et al. found that common variants in *NEFL* were associated with neuroblastoma susceptibility with 2101 neuroblastoma cases and 4202 controls of Caucasian ancestry as well as 459 neuroblastoma patients and 809 cancer-free controls of Italian descent [19]. They also discover a functional variant in the *CDKN1B* gene modifying neuroblastoma susceptibility [20]. Moreover, several other predisposing genes including *BARD1* [21], *FAS* and *FASL* [22], *XPG* [23], *HOTAIR* [24], and *ERCC1*/*XPF* [25] have been discovered through candidate gene approaches.

The human genome continuously suffers from DNA damages caused by exogenous (e.g., ultraviolet light, ionizing radiation chemicals) and endogenous (intracellular hydrolysis and metabolic by-products) factors. For example, the reactive oxygen species can give rise to oxidant-induced base lesions [26], which may eventually lead to genomic instability and increase tumor susceptibility if not repaired accurately. Hence, the DNA repair systems play important roles in maintaining the genomic integrity and cellular normal physiological function [27].

Base excision repair (BER) is one of the most important repair mechanisms for DNA lesions. It is widely accepted that BER is the primary pathway for repairing numerous oxidized and alkylated bases [28]. BER maintains genome integrity through recognition and excision of damaged bases. The protein product of the *apurinic/apyrimidinic endonuclease 1* (*APEX1*) gene is one of critical enzymes in this DNA repair pathway [29]. It is reasonable to speculate that functional single nucleotide polymorphisms (SNPs) in *APEX1* may affect DNA repair ability by affecting expression or function of the gene, which may further cause cell dysfunction, mutagenesis, and eventually tumorigenesis [30]. *APEX1* gene polymorphisms have been shown to associate with colorectal cancer [31], cervical cancer [32], and ovarian cancer [33]. However, no publications have reported the associations of *APEX1* gene polymorphisms with neuroblastoma risk. Therefore, we performed this three-center case-control study to assess such association in Chinese children.

## 2. Materials and Methods

### 2.1. Study Population

In the current three-center case-control research, a total of 469 histopathologically diagnosed neuroblastoma cases and 998 cancer-free controls of Chinese origin were recruited (Supplemental Table 1). Among them, 275 cases and 531 controls were recruited from Guangzhou [34–37], 118 cases and 281 controls from Zhengzhou [38–40], and the remaining 76 cases and 281 controls from Xi'an [41]. Informed consent was signed by the parents or guardians of all participants. The research protocol was authorized by the institutional review boards of corresponding institutions.

### 2.2. Polymorphism Selection and Genotyping

Potential functional polymorphisms in the *APEX1* gene were identified through the dbSNP database (http://www.ncbi.nlm.nih.gov/) and SNPinfo (http://snpinfo.niehs.nih.gov/) [42]. Briefly, we searched the SNPs located in the untranslated regions, two terminals, and exon of the *APEX1* gene. Additional selection criteria were referred from our previous publication [43]. Three SNPs (rs1130409 T>G, rs1760944 T>G, and rs3136817 T>C) in the *APEX1* gene were eventually selected. As shown in Supplemental Figure 1, there is no significant linkage disequilibrium (*R* ^2^ < 0.8) among these three included SNPs for Chinese Han subjects (*R* ^2^ = 0.130 between rs1760944 and rs3136817, *R* ^2^ = 0.076 between rs1760944 and rs1130409, and *R* ^2^ = 0.131 between rs3136817 and rs1130409). Genomic DNA was extracted from venous blood of participants applying the TIANamp Blood DNA Kit (TIANGEN Biotech Co. Ltd., Beijing, China). Then, the purified DNA samples were genotyped for the selected polymorphisms by the standard TaqMan real-time PCR [44–47]. To ensure the reliability of the results, 10% of the DNA samples were chosen randomly for a second-time genotyping. All duplicate samples receive a concordance rate of 100%.

### 2.3. Statistical Analysis

Deviation from the Hardy-Weinberg equilibrium (HWE) for the selected SNPs in all controls was checked by a goodness-of-fit *χ* ^2^ test. A two-sided chi-square test was used to compare distributions of demographics and allele frequencies between all cases and controls. By unconditional logistic regression analysis, odds ratios (ORs) and 95% confidence intervals (CIs) were calculated for assessing the association between *APEX1* polymorphisms and neuroblastoma risk. Furthermore, we conducted stratified analysis according to age, gender, tumor origin site, and clinical stage. All statistical analyses were performed by applying version 9.4 SAS software (SAS Institute, NC, USA). The *P* values < 0.05 were thought to be statistically significant.

## 3. Results

### 3.1. Associations between *APEX1* Polymorphisms and Neuroblastoma Risk

In general, this three-center case-control study contains 469 cases and 998 controls, of which genotyping was successfully performed in 469 cases and 997 controls. As shown in Table 1, the observed genotype frequencies of the three polymorphisms are consistent with HWE in control subjects (rs1130409: HWE = 0.190, rs1760944: HWE = 0.231, and rs3136817: HWE = 0.783). Unfortunately, after adjusting for age and gender, we failed to find significant association between the selected *APEX1* polymorphisms and neuroblastoma risk in the single locus analysis among combined subjects (Table 1) and among subjects from each center (Supplemental Table 2). We further assessed the combined effect of protective polymorphisms of *APEX1* gene on neuroblastoma risk. Subjects carrying 1-3 combined protective genotypes of *APEX1* have a lower risk of neuroblastoma when compared with those without a protective genotype, though not statistically significant (adjusted OR = 0.81, 95%CI = 0.64‐1.01, *P* = 0.064) (Table 1).

### 3.2. Stratification Analysis

To evaluate the effects of selected *APEX1* polymorphisms on neuroblastoma risk among different subgroups, stratified analysis was conducted based on the age, gender, site of tumor origin, and clinical stage (Table 2). Null correlations were found between two polymorphisms (rs1760944 T>G and rs3136817 T>C) and neuroblastoma risk. Interestingly, we detected that subjects with rs1130409 GG genotype have significantly decreased neuroblastoma risk in the male subgroup (adjusted OR = 0.67, 95%CI = 0.44‐0.995, *P* = 0.047) compared with the reference group. To assess the cumulative effects of protective genotypes among subgroups, we further performed a combined analysis. Results show that subjects harboring 1-3 protective genotypes had a significantly reduced neuroblastoma risk in the following subgroups: age > 18 months (adjusted OR = 0.70, 95% = 0.52‐0.94, *P* = 0.016) and males (adjusted OR = 0.64, 95%CI = 0.47‐0.87, *P* = 0.005).

## 4. Discussion

With an aim to investigate the effect of *APEX1* gene polymorphisms on neuroblastoma risk, we performed this current three-center case-control study in Chinese population. Neither rs1760944 T>G nor rs3136817T>C significantly modified neuroblastoma susceptibility. However, it is noteworthy that rs1130409 GG genotype was significantly associated with reduced neuroblastoma risk in the male subgroup.

The *APEX1* gene is mapped to chromosome 14q11.2-q12 and comprises five exons and four introns with 2.21 kb length. It encodes a nuclease acting as a rate-limiting enzyme in the BER pathway [48]. When exposed to endogenous and exogenous mutagens, apurinic/apyrimidinic (AP) sites are produced in DNA. *APEX1* can hydrolyze a 5′-phosphodiester bond at the AP site, thereby protecting cells against the accumulation of AP sites in DNA [49]. In addition, *APEX1* can act as 3′-phosphodiesterase which could hydrolyze 3′-blocking fragments of oxidized DNA, thus producing normal nucleotide 3′-droxyl terminals. These ends are requisite for DNA repair synthesis and ligation of gaps generated from single- or double-strand breaks [50, 51]. *APEX1* is endonuclease and phosphodiesterase in the BER pathway. Polymorphisms in the *APEX1* gene may alter gene expression so as to affect the activity of DNA repair and the accumulation of damaged DNA, eventually leading to carcinogenesis.

Variants in the *APEX1* gene are found to associate with susceptibility to several cancers, including cervical cancer [32], glioblastoma [52], bladder cancer [53], and lung cancer [49, 54]. To date, many SNPs in the *APEX1* gene have been identified [55]. Among them, polymorphisms rs1130409 T>G and rs1760944 T>G were extensively studied. Polymorphism rs1130409 T>G in the fifth exon leads to Asp148Glu residue transition in the carboxy-terminus but has no direct effect on DNA-binding and endonuclease activities of APEX1. However, polymorphism rs1760944 T>G in the promoter region may be a pathogenic SNP associated with cancer risk [54]. The rs1130409 has been widely explored in a wide spectrum of cancers, such as bladder cancer [53]. De Ruyck et al. suggested that rs1130409 T>G increased lung cancer risk in Caucasians [56], but Deng et al. found that this polymorphism reduced the susceptibility to lung cancer in Asians [57]. The rs1760944 T>G, one of the most frequently investigated SNPs in the *APEX1* gene, is also related to cancer susceptibility, such as nasopharyngeal carcinoma (NPC) [55] and glioblastoma [52]. Lo et al. suggested that patients with genotype rs1760944 GT or GG had lower lung cancer risk compared with those with rs1760944 TT [49]. One study performed by Kang et al. showed that the genotype of rs1760944 GG or GT may act as a protective genotype for breast cancer [58]. Li et al. found that genotype rs1760944 GG was associated with reduced risk of NPC compared with other genotypes, but the results did not reach statistical significance. More recently, one study conducted by Lu et al. confirmed that individuals with genotype rs1760944 GT or GG had significantly decreased NPC susceptibility than those with genotype rs1760944 TT [59]. These findings indicated that rs1760944 G allele may exert protective effects against several cancers. In addition, rs3136817 T>C is one of the polymorphisms in the *APEX1* gene which is investigated relatively less frequently. However, there are still some reports about association between polymorphism rs3136817 T>C and cancer risk. For instance, Zhu et al. showed that rs3136817 TC genotype of the *APEX1* gene was associated with lower risk of bladder cancer [60]. However, a research performed by Li et al. revealed null correlation between *APEX1* rs3136817 T>C and risk of severe radiation-induced pneumonitis after radiotherapy among patients with lung cancer [61]. A recent study conducted by Wang et al. indicated that the rs3136817 TC heterozygous genotype of *APEX1* has a potential protective effect for breast cancer, which may be owing to positive effect of rs3136817 TC genotype on DNA repair activity [62]. The conflicting association between *APEX1* gene polymorphisms and cancer risk may be explained by the differences in the sample sizes, race, cancer types, selection criteria for subjects, and environmental exposures. Therefore, the conclusion associated between *APEX1* polymorphisms and cancer risk should be specified in a particular cancer type and population.

Herein, we for the first time investigated the association between the *APEX1* gene SNPs and neuroblastoma risk. No significant association was found in the single locus analysis or combined analysis. Several reasons may help to explain the unexpected negative results: (1) the role of a polymorphism in cancer susceptibility might be tissue- and ethnicity-dependent and (2) the sample size of this study was too small to detect a real association. Although no significant effect was found in the overall analysis, age and gender may modify the results. In the stratified analysis, we found that rs1130409 GG genotype significantly decreased the risk of neuroblastoma in the male subgroup. To date, the rs1130409 T>G of the *APEX1* gene is the most extensively studied SNP. Moreover, we also found that the presence of 1-3 protective genotypes provided a protective effect for children > 18 months of age and boys. It should be noted that these significant results may be just chance findings in the stratification analysis because of relatively small sample size.

There are several accompanied limitations. First, although subjects in this current study were recruited from three independent hospitals, the sample size remains moderate, especially for the stratified analysis. As a result, the statistical powers were ineluctably whittled. Second, only three polymorphisms in the *APEX1* gene were investigated. Other potentially functional *APEX1* polymorphisms should be assessed. Third, the subjects involved in this study are Chinese; therefore, the conclusions obtained from this study may not apply to other ethnicities. Fourth, only genetic analysis was performed in neuroblastoma risk. Other environmental factors such as living environment, dietary habits, and childhood or parental exposure should be considered, as neuroblastoma is a heterogeneous disease with a complicated etiology.

## 5. Conclusions

In conclusion, our present data suggest that *APEX1* polymorphisms affect neuroblastoma susceptibility in a low-penetrance manner. Well-designed multicenter studies with larger sample size are needed to confirm our findings. Furthermore, functional validation should be performed to clarify the underlying mechanisms by which *APEX1* gene polymorphisms affect neuroblastoma susceptibility.

## Figures and Tables

**Table 1:** Association between *APEX1* gene polymorphisms and neuroblastoma risk.

Genotype	Cases (*N* = 469)	Controls (*N* = 997)^a^	*P* ^b^	Crude OR (95% CI)	*P*	Adjusted OR (95% CI)^c^	*P* ^c^
rs1130409 (HWE = 0.190)							
TT	175 (37.31)	340 (34.10)		1.00		1.00	
TG	216 (46.06)	467 (46.84)		0.90 (0.70-1.15)	0.389	0.90 (0.70-1.15)	0.390
GG	78 (16.63)	190 (19.06)		0.80 (0.58-1.10)	0.167	0.79 (0.58-1.09)	0.159
Additive			0.367	0.89 (0.77-1.04)	0.157	0.89 (0.76-1.04)	0.151
Dominant	294 (62.69)	657 (65.90)	0.230	0.87 (0.69-1.09)	0.230	0.87 (0.69-1.09)	0.226
Recessive	391 (83.37)	807 (80.94)	0.262	0.85 (0.63-1.13)	0.263	0.84 (0.63-1.13)	0.250
rs1760944 (HWE = 0.231)							
TT	155 (33.05)	334 (33.50)		1.00		1.00	
TG	230 (49.04)	470 (47.14)		1.05 (0.82-1.35)	0.674	1.06 (0.83-1.35)	0.665
GG	84 (17.91)	193 (19.36)		0.94 (0.68-1.29)	0.694	0.94 (0.68-1.29)	0.700
Additive			0.736	0.98 (0.84-1.15)	0.802	0.98 (0.84-1.15)	0.810
Dominant	314 (66.95)	663 (66.50)	0.864	1.02 (0.81-1.29)	0.864	1.02 (0.81-1.29)	0.854
Recessive	385 (82.09)	804 (80.64)	0.509	0.91 (0.69-1.21)	0.509	0.91 (0.69-1.21)	0.511
rs3136817 (HWE = 0.783)							
TT	396 (84.43)	815 (81.75)		1.00		1.00	
TC	67 (14.29)	172 (17.25)		0.80 (0.59-1.09)	0.158	0.80 (0.59-1.09)	0.163
CC	6 (1.28)	10 (1.00)		1.24 (0.45-3.42)	0.685	1.23 (0.44-3.41)	0.692
Additive			0.329	0.87 (0.66-1.14)	0.300	0.87 (0.66-1.14)	0.306
Dominant	73 (15.57)	182 (18.25)	0.205	0.83 (0.61-1.11)	0.206	0.83 (0.62-1.11)	0.211
Recessive	463 (98.72)	987 (99.00)	0.635	1.28 (0.46-3.54)	0.636	1.27 (0.46-3.52)	0.644
Combined effect of protective genotypes^d^							
0	305 (65.03)	598 (59.98)		1.00		1.00	
1-3	164 (34.97)	399 (40.02)	0.064	0.81 (0.64-1.01)	0.064	0.81 (0.64-1.01)	0.064

OR: odds ratio; CI: confidence interval; HWE: Hardy-Weinberg equilibrium. ^a^One was failed in genotyping. ^b^The *χ* ^2^ test for genotype distributions between neuroblastoma patients and cancer-free controls. ^c^Adjusted for age and gender. ^d^Protective genotypes were rs1130409 GG, rs1760944 GG, and rs3136817 TC/TT.

**Table 2:** Stratification analysis for association between *APEX1* gene genotypes and neuroblastoma susceptibility.

Variables	rs1130409 (case/control)		AOR (95% CI)^a^	*P* ^a^	rs1760944 (case/control)		AOR (95% CI)^a^	*P* ^a^	rs3136817 (case/control)		AOR (95% CI)^a^	*P* ^a^	Protective genotypes (case/control)		AOR (95% CI)^a^	*P* ^a^
	TT/TG	GG			TT/TG	GG			TT	TC/CC			0	1-3		
Age (month)																
≤18	138/319	31/71	1.01 (0.64-1.62)	0.958	135/314	34/76	1.04 (0.66-1.63)	0.874	139/325	30/65	1.09 (0.68-1.75)	0.733	101/234	68/156	1.01 (0.70-1.46)	0.949
>18	253/488	47/119	0.76 (0.52-1.09)	0.137	250/490	50/117	0.84 (0.58-1.21)	0.344	257/490	43/117	0.70 (0.48-1.02)	0.066	204/364	96/243	**0.70 (0.52-0.94)**	**0.016**
Gender																
Female	155/335	41/79	1.12 (0.73-1.71)	0.602	161/336	35/78	0.94 (0.60-1.46)	0.776	162/238	34/76	0.93 (0.60-1.46)	0.763	114/249	82/165	1.09 (0.77-1.53)	0.643
Male	236/472	37/111	**0.67 (0.44-0.995)**	**0.047**	224/468	49/115	0.89 (0.61-1.29)	0.527	234/477	39/106	0.75 (0.51-1.12)	0.166	191/349	82/234	**0.64 (0.47-0.87)**	**0.005**
Sites of origin																
Adrenal gland	136/807	26/190	0.80 (0.51-1.25)	0.323	134/804	28/193	0.87 (0.56-1.34)	0.521	137/815	25/182	0.83 (0.53-1.31)	0.430	108/598	54/399	0.75 (0.53-1.07)	0.111
Retroperitoneal	114/807	24/190	0.90 (0.56-1.44)	0.661	113/804	25/193	0.92 (0.58-1.45)	0.710	119/815	19/182	0.71 (0.43-1.18)	0.189	87/598	51/399	0.88 (0.61-1.27)	0.485
Mediastinum	101/807	20/190	0.85 (0.51-1.40)	0.516	100/804	21/193	0.88 (0.53-1.44)	0.603	101/815	20/182	0.88 (0.53-1.47)	0.633	80/598	41/399	0.77 (0.51-1.14)	0.187
Others	33/807	7/190	0.91 (0.40-2.09)	0.822	30/804	10/193	1.38 (0.66-2.88)	0.388	31/815	9/182	1.29 (0.61-2.76)	0.508	23/598	17/399	1.10 (0.58-2.09)	0.763
Clinical stage																
I+II+4s	192/807	41/190	0.91 (0.63-1.32)	0.607	192/804	41/193	0.89 (0.61-1.29)	0.523	197/815	36/182	0.81 (0.55-1.19)	0.283	148/598	85/399	0.85 (0.64-1.15)	0.293
III+IV	179/807	37/190	0.86 (0.58-1.27)	0.436	176/804	40/193	0.94 (0.64-1.37)	0.747	183/815	33/182	0.81 (0.54-1.22)	0.321	143/598	73/399	0.76 (0.56-1.04)	0.088

AOR: adjusted odds ratio; CI: confidence interval. ^a^Adjusted for age and gender, omitting the corresponding stratify factor.

## Data Availability

The data used to support the findings of this study are available from the corresponding author upon request.

## Abbreviations

GWAS: Genome-wide association study
BER: Base excision repair
*APEX1*: *Apurinic/apyrimidinic endonuclease 1*
SNP: Single nucleotide polymorphism
HWE: Hardy-Weinberg equilibrium
OR: Odds ratio
CI: Confidence interval.

## Supplementary Materials

Supplementary Materials Supplemental Table 1: frequency distribution of selected characteristics in cases and controls. Supplemental Table 2: association between APEX1 gene polymorphisms and neuroblastoma risk (divided subjects). Supplemental Figure 1: linkage disequilibrium analysis for the three selected polymorphisms in the APEX1 gene in the Han Chinese population consisting of CHS (Southern Han Chinese) and CHB (Han Chinese in Beijing, China) subjects using data from LDlink online software (https://ldlink.nci.nih.gov/?tab=ldmatrix).
