# Supplementary material for: APEX1 Polymorphisms and Neuroblastoma Risk in Chinese Children: A Three-Center Case-Control Study
Source: Oxid Med Cell Longev. 2019 Jun 25;2019:5736175. doi: 10.1155/2019/5736175 (PMC6614964; doi:10.1155/2019/5736175)
Supplement: Supplementary Materials — Supplemental Table 1: frequency distribution of selected characteristics in cases and controls. Supplemental Table 2: association between APEX1 gene polymorphisms and neuroblastoma risk (divided subjects). Supplemental Figure 1: linkage disequilibrium analysis for the three selected polymorphisms in the APEX1 gene in the Han Chinese population consisting of CHS (Southern Han Chinese) and CHB (Han Chinese in Beijing, China) subjects using data from LDlink online software (https://ldlink.nci.nih.gov/?tab=ldmatrix). [file 5736175.f1.pdf]

Supplemental Table 1. Frequency distribution of selected characteristics in cases and controls

Variables	Cases (n=469)		Controls (n=998)		*P* [a]
	No.	%	No.	%	
Age range, month	0.00-132.00		0.03-156.00		0.263
Mean ± SD	34.07±27.60		32.89±27.43		
≤18	169	36.03	390	39.08	
>18	300	63.97	608	60.92	
Gender					0.911
Female	196	41.79	414	41.48	
Male	273	58.21	584	58.52	
INSS stages					
I	133	28.42	/	/	
II	100	21.37	/	/	
III	69	14.74	/	/	
IV	147	31.41	/	/	
4s	11	2.35	/	/	
NA	8	1.71	/	/	
Sites of origin					
Adrenal gland	162	34.54	/	/	
Retroperitoneal region	138	29.42	/	/	
Mediastinum	121	25.80	/	/	
Other region	40	8.53	/	/	
NA	8	1.71	/	/	

SD, standard deviation; NA, not available.

[a] Two-sided χ^2 test for distributions between neuroblastoma cases and cancer-free controls.

Supplemental Table 2. Association between *APEX1* gene polymorphisms and neuroblastoma risk (Divided subjects)

Genotype	Guangdong province				Henan province				Shaanxi province			
	Cases (N=275)	Controls (N=531)	AOR (95% CI) [a]	P [a]	Cases (N=118)	Controls (N=281) [b]	AOR (95% CI) [a]	P [a]	Cases (N=76)	Controls (N=186)	AOR (95% CI) [a]	P [a]
rs1130409 T>G												
TT	108 (39.27)	210 (39.55)	1.00		37 (31.36)	77 (27.50)	1.00		30 (39.47)	53 (28.49)	1.00	
TG	129 (46.91)	227 (42.75)	1.10 (0.80-1.51)	0.547	56 (47.46)	152 (54.29)	0.77 (0.46-1.26)	0.296	31 (40.79)	88 (47.31)	0.62 (0.34-1.15)	0.129
GG	38 (13.82)	94 (17.70)	0.78 (0.50-1.22)	0.282	25 (21.19)	51 (18.21)	0.97 (0.52-1.81)	0.912	15 (19.74)	45 (24.19)	0.59 (0.28-1.22)	0.154
Additive			0.93 (0.76-1.14)	0.486			0.96 (0.70-1.31)	0.779			0.75 (0.52-1.08)	0.120
Dominant	167 (60.73)	321 (60.45)	1.01 (0.75-1.36)	0.952	81 (68.64)	203 (72.50)	0.82 (0.51-1.31)	0.401	46 (60.53)	133 (71.51)	0.61 (0.35-1.07)	0.085
Recessive	237 (86.18)	437 (82.30)	0.74 (0.50-1.12)	0.157	93 (78.81)	229 (81.79)	1.14 (0.66-1.97)	0.633	61 (80.26)	141 (75.81)	0.76 (0.40-1.47)	0.420
rs1760944 T>G												
TT	101 (36.73)	186 (35.03)	1.00		34 (28.81)	93 (33.21)	1.00		20 (26.32)	55 (29.57)	1.00	
TG	128 (46.55)	245 (46.14)	0.96 (0.70-1.33)	0.816	62 (52.54)	125 (44.64)	1.38 (0.84-2.27)	0.210	40 (52.63)	100 (53.76)	1.11 (0.59-2.09)	0.748
GG	46 (16.73)	100 (18.83)	0.85 (0.56-1.31)	0.462	22 (18.64)	62 (22.14)	0.99 (0.53-1.85)	0.970	16 (21.05)	31 (16.67)	1.43 (0.65-3.15)	0.380
Additive			0.93 (0.76-1.14)	0.489			1.03 (0.76-1.39)	0.863			1.19 (0.80-1.76)	0.398
Dominant	174 (63.27)	345 (64.97)	0.93 (0.69-1.26)	0.642	84 (71.19)	187 (66.79)	1.25 (0.78-2.00)	0.358	56 (73.68)	131 (70.43)	1.19 (0.65-2.16)	0.581
Recessive	229 (83.27)	431 (81.17)	0.87 (0.59-1.28)	0.482	96 (81.36)	218 (77.86)	0.81 (0.47-1.40)	0.456	60 (78.95)	155 (83.33)	1.33 (0.68-2.61)	0.404
rs3136817 T>C												
TT	232 (84.36)	443 (83.43)	1.00		97 (82.20)	230 (82.14)	1.00		67 (88.16)	142 (76.34)	1.00	
TC	40 (14.55)	85 (16.01)	0.90 (0.60-1.35)	0.600	18 (15.25)	47 (16.79)	0.90 (0.50-1.63)	0.729	9 (11.84)	40 (21.51)	0.48 (0.22-1.05)	0.065
CC	3 (1.09)	3 (0.56)	1.87 (0.37-9.34)	0.448	3 (2.54)	3 (1.07)	2.33 (0.46-11.78)	0.308	0 (0.00)	4 (2.15)	/	/
Additive			0.97 (0.67-1.41)	0.872			1.07 (0.65-1.75)	0.792			0.43 (0.21-0.91)	0.026
Dominant	43 (15.64)	88 (16.57)	0.93 (0.62-1.38)	0.720	21 (17.80)	50 (17.86)	0.99 (0.56-1.74)	0.962	9 (11.84)	44 (23.66)	0.44 (0.20-0.94)	0.035
Recessive	272 (98.91)	528 (99.44)	1.90 (0.38-9.49)	0.436	115 (97.46)	277 (98.93)	2.37 (0.47-11.96)	0.297	76 (100.00)	182 (97.85)	/	/
Combined effect of protective genotypes [c]												
0	184 (66.91)	337 (63.47)	1.00		75 (63.56)	160 (57.14)	1.00		46 (60.53)	101 (54.30)	1.00	
1-3	91 (33.09)	194 (36.53)	0.87 (0.64-1.18)	0.358	43 (36.44)	120 (42.86)	0.75 (0.48-1.17)	0.203	30 (39.47)	85 (45.70)	0.78 (0.45-1.33)	0.357

AOR, adjusted odds ratio; CI, confidence interval.

[a] Adjusted for age and gender.

[b] One was failed in genotyping.

[c] Protective genotypes were rs1130409 GG, rs1760944 GG and rs3136817 TC/TT derived from combined subjects.

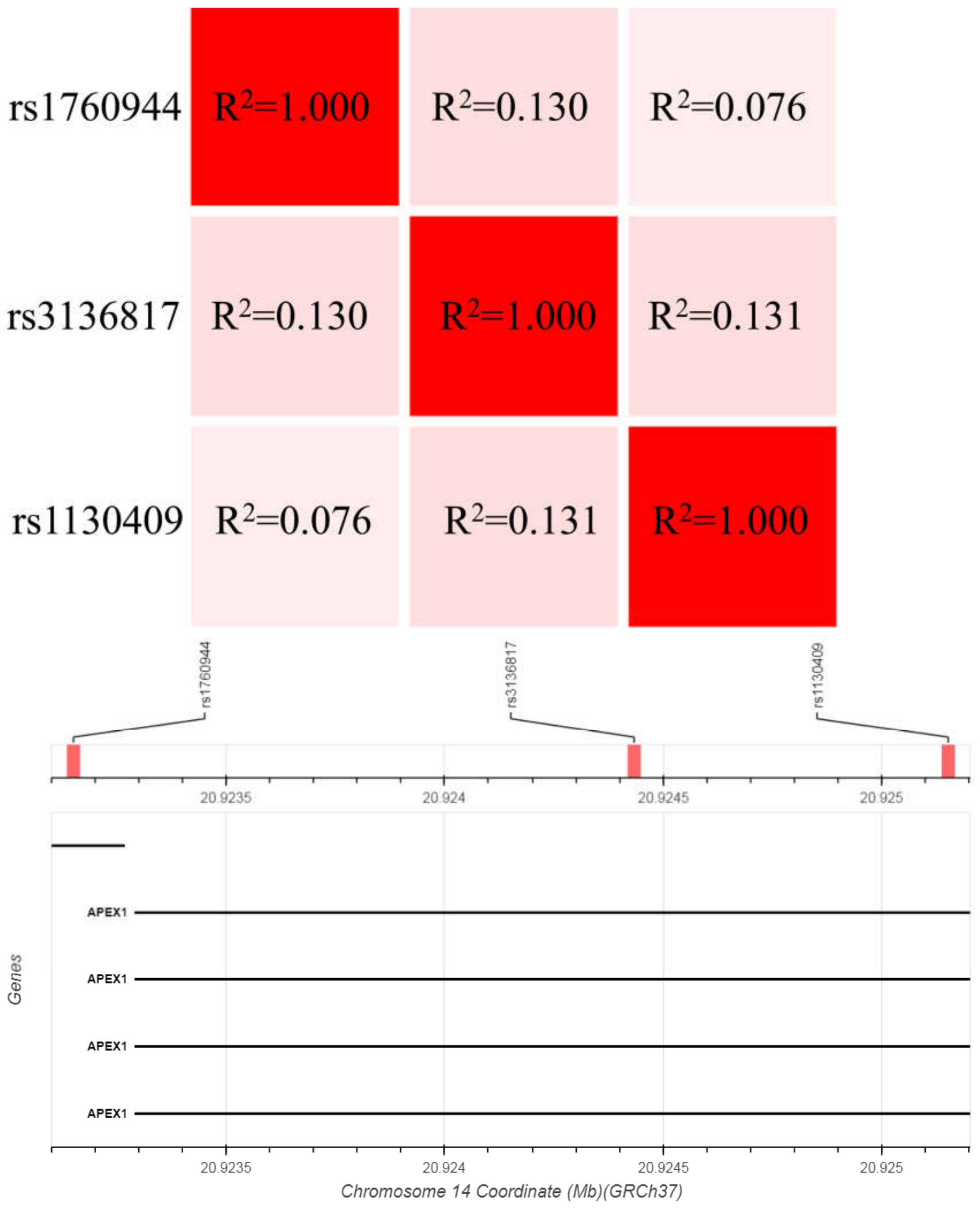

Supplemental Figure 1. Linkage disequilibrium analysis for the three selected polymorphisms in *APEX1* gene in Han Chinese population consisting of CHS (Southern Han Chinese) and CHB (Han Chinese in Beijing, China) subjects using data from LDlink online software (https://ldlink.nci.nih.gov/?tab=ldmatrix).
